# Supplementary material for: Crystal Structures of Bacterial Pectin Methylesterases Pme8A and PmeC2 from Rumen Butyrivibrio
Source: Int J Mol Sci. 2023 Sep 6;24(18):13738. doi: 10.3390/ijms241813738 (PMC10530356; doi:10.3390/ijms241813738)
Supplement: Supplementary file 1 [file ijms-24-13738-s001.zip › ijms-2583828-supplementary.pdf]

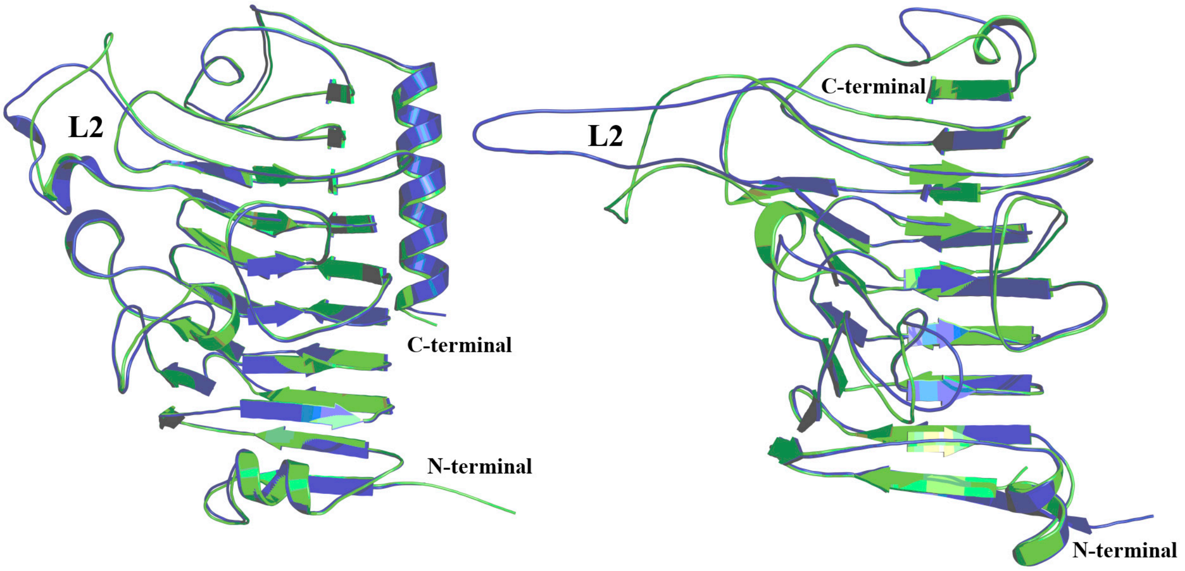

Figure S1: The structures of Pme8A (8TNE, left) and PmeC2 (8TMS, right). The refined crystal structures are in blue and the predicted Alphafold2 models are in green. The main body of enzymes are remarkably identical with respect to secondary structure and side chain rotamer assignment. However, large changes are observed with the alpha helical arrangement of the L2 loop for Pme8A. While for PmeC2, larger movements were observed for the solvent-exposed loops that connect and extend from the beta strands of the enzyme such as the residues on the L2 loop and the C-terminus
